# Screening for participants in the ISCHEMIA trial: Implications for clinical research

**DOI:** 10.1017/cts.2022.428

**Published:** 2022-07-13

**Authors:** Fatima Rodriguez, Judith S. Hochman, Yifan Xu, Harmony R. Reynolds, Jeffrey S. Berger, Stavroula Mavromichalis, Jonathan D. Newman, Sripal Bangalore, David J. Maron

**Affiliations:** 1 Division of Cardiovascular Medicine and the Cardiovascular Institute, Stanford University, Stanford, CA, USA; 2 Stanford Prevention Research Center, Department of Medicine, Stanford University, School of Medicine, Stanford, CA, USA; 3 New York University, Grossman School of Medicine, New York, NY, USA

**Keywords:** Clinical trials, disparities, enrollment, screening, stable ischemic heart disease

## Abstract

The International Study of Comparative Health Effectiveness with Medical and Invasive Approaches (ISCHEMIA) found that there was no statistical difference in cardiovascular events with an initial invasive strategy as compared with an initial conservative strategy of guideline-directed medical therapy for patients with moderate to severe ischemia on noninvasive testing. In this study, we describe the reasons that potentially eligible patients who were screened for participation in the ISCHEMIA trial did not advance to enrollment, the step prior to randomization. Of those who preliminarily met clinical inclusion criteria on screening logs submitted during the enrollment period, over half did not participate due to physician or patient refusal, a potentially modifiable barrier. This analysis highlights the importance of physician equipoise when advising patients about participation in randomized controlled trials.

Participant recruitment in randomized controlled trials is often a challenge in terms of the time and cost required to complete a trial and the representativeness of the sample that is included. The objective of this report is to describe the reasons that potentially eligible participants who were screened did not advance to enrollment, the step prior to randomization in the International Study of Comparative Health Effectiveness with Medical and Invasive Approaches (ISCHEMIA) [1].

**Table 1.** Characteristics of patients screened for the ISCHEMIA trial

	Screened	Met clinical exclusion	Clinically eligible but excluded			Consented for enrollment
			Physician refused	Patient refused	Other^*^	
	*n* = 26,254	*n* = 12,325	*n* = 3,039	*n* = 2,672	*n* = 5,452	*n* = 2,766
Age, Median (Q1, Q3)	66 (58, 73)	67 (59, 74)	67 (59, 74)	66 (59, 73)	65 (57, 72)	63 (56, 69)
Female *n* (row %)^†^	7665/26072 (29.4%)	3503/7665 (45.7%)	955/4162 (22.9%)	830/4162 (19.9%)	1659/4162 (39.9%)	718/4162 (17.2%)
Male *n* (row %)^†^	18407/26072 (70.6%)	8762/18407 (47.6%)	2065/9645 (21.4%)	1821/9645 (18.9%)	3722/9645 (38.6%)	2037/9645 (21.1%)
Region, *n* (row %)						
Asia	2810/26254 (10.7%)	490/2810 (17.4%)	217/2320 (9.4%)	494/2320 (21.3%)	695/2320 (30.0%)	914/2320 (39.4%)
Europe	4230/26254 (16.1%)	2054/4230 (48.6%)	310/2176 (14.2%)	469/2176 (21.6%)	706/2176 (32.4%)	691/2176 (31.8%)
Latin America	1274/26254 (4.9%)	777/1274 (61%)	107/497 (21.5%)	65/497 (13.1%)	143/497 (28.8%)	182/497 (36.6%)
North America	16870/26254 (64.3%)	8261/16870 (49%)	2342/8609 (27.2%)	1566/8609 (18.2%)	3799/8609 (44.1%)	902/8609 (10.5%)
Other	1070/26254 (4.1%)	743/1070 (69.4%)	63/327 (19.3%)	78/327 (23.9%)	109/327 (33.3%)	77/327 (23.5%)

* Other patients were excluded for reasons including not enough clinical information to determine if subject was suitable for inclusion (*n* = 496), could not be contacted (*n* = 513), went straight to angiography (*n* = 526), opted for medical therapy (*n* = 10), ischemia not verified by adjudication (*n* = 19), and unknown reasons (*n* = 3888).

† A total of 182 patients screened were missing sex information.

The ISCHEMIA trial compared an initial invasive stragegy added to guideline-directed medical therapy with a conservative management strategy of guideline-directed medical therapy alone in patients with stable ischemic heart disease with significant ischemia. Patients with stable ischemic heart disease were selected for enrollment based on local site interpretation of moderate or severe ischemia on noninvasive stress testing [2]. Key clinical exclusion criteria were recent acute coronary syndrome, unprotected left main stenosis of at least 50%, left ventricular systolic function <35%, New York Heart Association Class III or IV heart failure, estimated glomerular filtration rate (eGFR) <30 ml/min, or unacceptable angina despite maximal medical therapy. Eligible patients who consented for enrollment were randomized to an initial invasive strategy (*n* = 2,588) versus an initial conservative strategy (*n* = 2,591). Almost a quarter of those randomized were women (23%). After a median follow-up of 3.2 years, an initial invasive strategy did not reduce major adverse cardiovascular events as compared with initial conservative management (13.3% vs. 15.5%, *P* = 0.34), although patients with angina at baseline assigned to the invasive strategy had greater improvement in quality of life [1, 3].

## Methods

Potential participants were identified and recruited from stress testing laboratories. The ischemia eligibility criteria were at least moderate ischemia on a stress imaging test or severe ischemia on a non-imaging exercise tolerance test as defined by the protocol and interpreted by the site.

From January 2012 to February 2018, we asked sites to complete screening logs of consecutive patients who underwent stress testing and met ischemia eligibility criteria and to submit them to the Clinical Coordinating Center (CCC) every month. This activity was not funded, and sites were not required to submit logs over this entire period. Sites that enrolled at least one participant per month over the previous 3 months were not asked to submit screening logs. The information provided on the log included the total number of stress tests performed in the past month at the site’s primary stress laboratory. Data entered for patients who met ischemia eligibility criteria were age, sex, and whether the patient provided consent or was excluded due to meeting an exclusion criterion, along with the reason for exclusion. Refusal by the patient or the patient’s physician was also documented, but not the reason for such refusal. As of January 1, 2017, submission of screening logs to the CCC became optional for all sites.

This analysis includes patients entered on screening logs for which information about clinical eligibility was available. We computed descriptive statistics as frequencies and percentages for categorical variables and medians and interquartile ranges for continuously measured variables.

## Results

A total of 380 sites in 38 countries were eligible to screen and enroll participants. Among these, 339 sites enrolled participants and 308 sites (81%) submitted screening logs. A total of 4,014 screening logs collected from 308 sites between January 2012 and February 2018 (22,792 site months) were considered in this analysis, with a total of 28,324 patients entered. We excluded 1,987 patients for whom the reason for exclusion was not documented, 75 patients from sites only enrolling in ISCHEMIA-Chronic Kidney Disease, and 8 with input errors, yielding a sample of 26,254 patients with moderate or severe ischemia on stress testing. Of these, a total of 12,325 met protocol-defined clinical exclusion criteria and 2,766 were consented and enrolled (Table 1). Among the remaining 11,163 apparently clinically eligible but excluded patients, 3,039 (27.2%) were excluded due to physician refusal, 2,672 (23.9%) were excluded due to patient refusal, and 5,452 were excluded for various other reasons. Sites reported that some patients were excluded due to difficulty with patient contact (*n* = 513, 4.6%) or referral to angiography before consent could be obtained (*n* = 526, 4.7%). Few patients (*n* = 43, 0.2% of those screened) with eGFR between 30 and 59 ml/min were excluded because of physician suspicion of unprotected left main disease. Of all women screened, 9.4% were consented for trial enrollment compared with 11.1% of all men. A more pronounced sex difference was observed among those in this sample who appeared clinically eligible at screening who subsequently agreed to participate in the trial: 17.2% of women consented versus 21.1% of men (*P* < 0.001).

Participants from study sites located in Asia were more likely to consent for trial enrollment (39.4%), whereas those from North America were least likely to enroll (10.5%). Among clinically eligible patients who were excluded, patient refusal was highest in Other regions (23.9%) and lowest in Latin America (13.1%). Physician refusal was highest in North America (27.2%) and lowest in Asia (9.4%).

## Discussion

In this analysis, we report the rates of physician and patient refusal for trial enrollment within a sample of patients who were screened for ISCHEMIA based on moderate or severe ischemia on stress testing. Of those who apparently met clinical inclusion criteria on preliminary screening, over half did not participate due to physician or patient refusal. This rate of physician and patient refusal was disappointing and illustrates an important challenge to recruiting patients to participate in clinical trials. When there is uncertainty about the efficacy of an intervention, the best method to establish evidence is to conduct a randomized controlled trial. Physicians must have equipoise when optimal management is uncertain and explain to patients that there is a gap in evidence to define optimal management of their condition. Indeed, one could question the ethics of practicing without evidence or preventing a patient from being informed about or participating in a clinical trial when there is uncertainty regarding the best management strategy. In the case of ISCHEMIA, despite community equipoise whether revascularization reduces clinical event rates in patients with stable ischemic heart disease [4], we encountered challenges to enrolling apparently eligible patients with moderate or severe ischemia into a trial with a 50% chance of being randomized to a conservative (i.e., non-revascularization) management strategy. These decisions are undoubtedly influenced by preexisting patient and physician beliefs regarding the risks and benefits of the treatment strategies tested. Notably, sites in the North American region had the lowest rates of enrollment and were more likely to have physicans refuse study participation than other study regions.

Data show that women are less likely to undergo stress testing, a phenomenon in part attributable to implicit sex bias of cardiologists [5]. ISCHEMIA also found that women who were screened for study participation were less likely to consent as compared with men, with sex-based disparities more pronounced among clinically eligible participants [6]. This suggests that the observed sex-specific disparities are not fully explained by differences in the prevalence of obstructive coronary disease between men and women with similar degrees of ischemia. Female sex has been associated with lower clinical trial participation rates in other cardiovascular and stroke randomized trials, threatening the generalizability of study results [7, 8]. Similar to our study that documents that women were more likely to decline study participation, one study found that women were 15% less willing to participate in cardiovascular disease prevention trials, perceiving a greater chance of harm than benefit from trial participation [9]. Given the high burden of cardiovascular morbidity and mortality in women, increasing representation of women in practice-informing clinical trials is of paramount importance. Analyses from ISCHEMIA have already demonstrated sex differences between participants in terms of angina severity, anatomic severity of disease, degree of ischemia, and risk factor control [6, 10].

A limitation of this study is that the screening log sample does not reflect all patients screened, enrolled, and randomized into the trial. Screening logs were completed intermittently at only 81% of enrolling sites and were skewed toward lower enrolling sites in line with the purpose of screening logs as a tool to assess and act upon enrollment challenges. Two-thirds of those screened in this cohort had clinical exclusion criteria. The screening log did not assess race, ischemia severity (moderate vs. severe), angina severity, the reasons for physician and patient refusal, or clinical outcomes for excluded patients. The proportion of patient and physician refusals is subject to uncertainty as the assessment of clinical eligibility was preliminary, for example, details were not pursued further if there was a refusal. It is possible that the reasons for refusal could have biased the sample in way that affected trial results.

In conclusion, analyses from screening logs from ISCHEMIA, submitted by sites over varying durations of the enrollment period, demonstrate that two-thirds of patients screened had a clinical exclusion criterion. Among apparently eligible patients excluded from trial enrollment, patient and physician refusal accounted for over half of exclusions, and women were less likely than men to agree to participate. These findings underscore the importance of physician equipoise when advising patients about participation in randomized controlled trials. More work is also needed to dismantle reasons for sex-based differences in cardiovascular clinical trial participation.

## References

[1] Maron DJ , Hochman JS , Reynolds HR , et al. Initial invasive or conservative strategy for stable coronary disease. New England Journal of Medicine 2020; 382(15): 1395–1407. 32227755 10.1056/NEJMoa1915922 PMC7263833

[2] ISCHEMIA Trial Research Group, Maron DJ , Hochman JS , et al. International study of comparative health effectiveness with medical and invasive approaches (ISCHEMIA) trial: Rationale and design. American Heart Journal 2018; 201: 124–135. 29778671 10.1016/j.ahj.2018.04.011 PMC6005768

[3] Spertus JA , Jones PG , Maron DJ , et al. Health-Status outcomes with invasive or conservative care in coronary disease. New England Journal of Medicine 2020; 382(15): 1408–1419. 32227753 10.1056/NEJMoa1916370 PMC7261489

[4] Stone GW , Hochman JS , Williams DO , et al. Medical therapy with versus without revascularization in stable patients with moderate and severe ischemia: The case for community equipoise. Journal of the American College of Cardiology 2016; 67(1): 81–99. 26616030 10.1016/j.jacc.2015.09.056 PMC5545795

[5] Daugherty SL , Blair IV , Havranek EP , et al. implicit gender bias and the use of cardiovascular tests among cardiologists. Journal of American Heart Association 2017; 6(12): e006872. 10.1161/JAHA.117.006872 PMC5779009 29187391

[6] Reynolds HR , Shaw LJ , Min JK , et al. Association of sex with severity of coronary artery disease, ischemia, and symptom burden in patients with moderate or severe ischemia: Secondary analysis of the ISCHEMIA randomized clinical trial. JAMA Cardiology 2020; 5(7): 773–786. 32227128 10.1001/jamacardio.2020.0822 PMC7105951

[7] Martin SS , Ou FS , Newby LK , et al. Patient- and trial-specific barriers to participation in cardiovascular randomized clinical trials. Journal of the American College of Cardiology 2013; 61(7): 762–769. 23410547 10.1016/j.jacc.2012.10.046

[8] O’Neill ZR , Deptuck HM , Quong L , et al. Who says “no” to participating in stroke clinical trials and why: An observational study from the Vancouver Stroke Program. Trials 2019; 20(1): 313. 31151483 10.1186/s13063-019-3434-0 PMC6545028

[9] Ding EL , Powe NR , Manson JE , Sherber NS , Braunstein JB Sex differences in perceived risks, distrust, and willingness to participate in clinical trials: A randomized study of cardiovascular prevention trials. Archives of Internal Medicine 2007; 167(9): 905–912. 17502531 10.1001/archinte.167.9.905

[10] Newman JD , Alexander KP , Gu X , et al. Baseline predictors of low-density lipoprotein cholesterol and systolic blood pressure goal attainment after 1 year in the ISCHEMIA trial. Circulation Cardiovascular Quality and Outcomes 2019; 12(11): e006002. 31718297 10.1161/CIRCOUTCOMES.119.006002 PMC7157834

